# Maternal Resveratrol Treatment Re-Programs and Maternal High-Fat Diet-Induced Retroperitoneal Adiposity in Male Offspring

**DOI:** 10.3390/ijerph17082780

**Published:** 2020-04-17

**Authors:** Ti-An Tsai, Chang-Ku Tsai, Li-Tung Huang, Jiunn-Ming Sheen, Mao-Meng Tiao, You-Lin Tain, Chih-Cheng Chen, I-Chun Lin, Yun-Ju Lai, Ching-Chou Tsai, Yu-Ju Lin, Hong-Ren Yu

**Affiliations:** 1 Department of Pediatrics, Chang Gung Memorial Hospital-Kaohsiung Medical Center, Graduate Institute of Clinical Medical Science, Chang Gung University College of Medicine, Kaohsiung 833, Taiwan; tiantsai@cgmh.org.tw (T.-A.T.); wind518@cgmh.org.tw (C.-K.T.); litung.huang@gmail.com (L.-T.H.); ray.sheen@gmail.com (J.-M.S.); pc006581@yahoo.com.tw (M.-M.T.); tainyl@hotmail.com (Y.-L.T.); charllysc@adm.cgmh.org.tw (C.-C.C.); uc22@adm.cgmh.org.tw (I.-C.L.); 2 Department of Obstetrics and Gynecology, Chang Gung Memorial Hospital-Kaohsiung Medical Center, Kaohsiung 833, Taiwan; lusionbear@hotmail.com (Y.-J.L.); nickcctsai@yahoo.com.tw (C.-C.T.); lyu015@cgmh.org.tw (Y.-J.L.)

**Keywords:** prenatal, high-fat diet, resveratrol, adipose tissue, leptin

## Abstract

Obesity during pregnancy increases the risk of cardiovascular problems, diabetes, asthma, and cognitive impairments, affecting the offspring. It is important to reduce the negative effects of obesity and high-fat (HF) diet during pregnancy. We employed a rat model of maternal HF diet to evaluate the possible de-programming effects of resveratrol in rodent male offspring with maternal HF diet/obesity. Male rat offspring were randomized into four groups: maternal control diet/postnatal control diet, maternal HF diet/postnatal control diet, maternal control diet plus maternal resveratrol treatment/postnatal control diet, and maternal HF diet plus maternal resveratrol treatment/postnatal control diet. Maternal HF diet during pregnancy plus lactation resulted in retroperitoneal adiposity in the male offspring. Maternal resveratrol treatment re-programmed maternal HF exposure-induced visceral adiposity. Offspring that received prenatal HF diet showed higher leptin/soluble leptin receptor (sOB-R) ratio than offspring that received prenatal control diet. Maternal resveratrol treatment ameliorated maternal HF exposure-induced increase in leptin/sOB-R ratio and altered the expression of genes for crucial fatty acid synthesis enzymes in the offspring. Thus, maternal resveratrol administration reduces retroperitoneal adiposity in rat offspring exposed to prenatal HF diet/obesity and could be used to ameliorate negative effects of maternal HF diet in the offspring.

## 1. Introduction

Malnutrition in women during pregnancy and/or lactation can have long-term adverse effects on the energy homeostasis of the offspring [1]. Both low and high birth weights of offspring can predispose to obesity in adolescence [2]. In addition to nutritional deficiency during pregnancy, maternal obesity has become a global health problem in developed countries. More than 30% of childbearing age women are morbidly obese according to this study [3]. Several previous studies have provided strong evidence of the negative effects of maternal obesity on the offspring. Emotional dysregulation, cognitive impairments, and lack of attention have been consistently observed in children born to mothers with high BMI and excess weight gain at pregnancy [4,5,6,7]. Furthermore, obesity during pregnancy is linked with an increased risk of coronary heart disease, diabetes, stroke, and asthma affecting the adult offspring [8,9,10]. Since maternal obesity has a long-term and profound impact on public health, there is a genuine need to study the potential pathological mechanisms by which it can affect the offspring, and to find an effective intervention to reverse the adverse effects of obesity in pregnant women and offspring.

The Developmental Origins of Health and Disease (DOHaD) concept suggests that the development of multiple tissues in the progeny can be imprinted by adverse perinatal environment and may determine physical condition and illness in later life. Therefore, nutritional supply to the fetus or the newborn offspring may be a key determinant affecting the metabolic homeostasis of an individual [1,2]. The hypothalamus–adipose axis, which integrates peripheral insulin, leptin, and nutritional information, is most likely involved in the developmental programming of the metabolic pathways in the individual [2]. The DOHaD also suggests prevention of metabolic dysregulation through re-programming, a strategy that has the potential to overcome current treatment options available only at adulthood, thereby providing therapeutic strategies in the early life of an individual [11].

In animal studies, maternal overnutrition or obesity was induced by feeding the dams with high-fat (HF) or high-energy diets during gestation and/or lactation stage [2]. Obesity in the offspring born to overfed or obese dams illustrates the underlying mal-programming of the appetite-regulating system in the hypothalamus [12]. Central leptin resistance was also observed in offspring with overfed or obese rodent mothers, showing higher hypothalamic SOCS3 activation and a blunted down-stream signaling of leptin hormone [13].

Supplements, such as folic acid, glycine, vitamin D, and n-3 fatty acids, when present in the maternal diet have the potential to reduce the adverse consequences of perinatal programming [2,14]. Resveratrol is a polyphenolic compound, a naturally occurring phytoalexin produced by several plants during stress. It inhibits platelet aggregation, endothelial function, inflammation, oxidative stress, carcinogenesis, and obesogenic functions [15,16,17]. Resveratrol intake during pregnancy plus lactation decreases body weight and fat tissue content in the offspring of dams on a HF diet compared with those offspring with maternal low-fat diet [18]. Resveratrol administration to the offspring can also rescue adiposity and leptin resistance predisposed by prenatal and postnatal HF exposure [19]. In this study, we tried to evaluate the possible de-programming mechanisms of maternal resveratrol administration in the male offspring of dams with HF diet/obesity.

## 2. Materials and Methods

### 2.1. Rats and Experimental Design

Virgin Sprague-Dawley (SD) rats were purchased from BioLASCO (BioLASCO Taiwan Co., Ltd., Taipei, Taiwan) and maintained according to the guide of the Association for Assessment and Accreditation of Laboratory Animal Care International. Maternal HF diet or control diet with or without resveratrol were employed to investigate the effects of maternal resveratrol intake on reduction of adverse effects of maternal HF diet and obesity. Female rats were weight-matched and randomized to accept either a regular control diet (ND; Fwusow Taiwan Co., Ltd., Taichung, Taiwan) or HF diet (D12331, Research Diets, Inc., New Brunswick, NJ, USA; 58% fat) ad libitum from 8 weeks age before mating, total gestational stage, and through lactation. Dams that received HF diet showed heavier body weight than those on regular control diet after one week (Supplementary Figure S1). For resveratrol intervention, 50 mg/L of resveratrol in the water was prescribed from pregnancy to lactation, as previously reported [20]. The male rats were coped with female rats until gravidity was identified by examining vaginal plug. After confirmation of pregnancy, the pregnant female rats were randomly appointed to four groups. A total of 20 female rats were mated to deliver 12 L. Their offspring were weaned at 3 weeks of postnatal age following which they were put on a control diet until they were 4 months old. Male offspring were enrolled in the subsequent experiments to avoid sex-dependent effects. Thus, four experimental groups of male offspring were designated as: maternal control diet/postnatal control diet (CC), maternal HF diet/postnatal control diet (HC), maternal control diet plus maternal resveratrol treatment/ postnatal control diet (CRC), and maternal HF diet plus maternal resveratrol treatment/ postnatal control diet (HRC). All the animals are from one experiment. Supplementary Figure S1 shows the body weight change of dams on regular control diet or HF diet for 8 weeks. Dams on HF diet for 8 weeks were heavier than those on control diet after one month. This study was conducted according to the recommendations of the Guide for the Care and Use of Laboratory Animals of the National Institutes of Health, and with the approval of the Institutional Animal Care and Use Committee of Chang Gung Memorial Hospital-Kaohsiung Medical Center (No. 2017032704).

### 2.2. Test for Intraperitoneal Glucose Tolerance

Blood samples were collected at five time-points after 8 *h* of fasting for the intraperitoneal glucose tolerance test: before, and 15, 30, 60, and 120 minutes after injection of glucose (2 g/kg) by intraperitoneal injection. The enzymatic (hexokinase) method was sued for plasma glucose determination.

### 2.3. Tissue Collection and Blood Sampling

The body weights (BW) were measured every month for the offspring until they were 4 months old. At the age of 4 months, the four groups of offspring were weighed and euthanized by Zoletil^®^ and xylazine administration [21]. Plasma and adipose tissue were collected as previously reported [21]. Plasma triglyceride and high-density lipoprotein (HDL) were determined using an automated clinical chemistry analyzer (FUJIFILM, FUJI Dry-Chem 4000i, Tokyo, Japan). Plasma leptin levels were determined by ELISA (Biovendor, Brno, Czech Republic) with 1.8% and 4.4% coefficient of variability (CV) for intra-assay and inter-assay, respectively. Plasma soluble leptin receptor (sOB-R) was determined by ELISA (BlueGene, Shanghai, China) with 4.2% and 6.6%CV for intra-assay and inter-assay, respectively.

### 2.4. Histological Examination

The adipose tissues were harvested and stored in saline on ice immediately after the animals were euthanized. After fixing in 10% formalin neutral buffer solution pH 7·4 (Wako Junyaku, Osaka, Japan), four-micrometer-thick sections were prepared and stained with hematoxylin and eosin (H&E) for morphometric interpretation. A mounted digital camera (Nikon, NY, USA) under a 10× objective of a Nikon Eclipse E600 microscope was used to capture images.

### 2.5. Western Blot Analysis

Western blot was conducted as previously described [22]. In brief, samples were lysed in ice-cold RIPA buffer with a protease and phosphatase inhibitor cocktail. After the concentrations of samples were checked, fifty micrograms of each sample were boiled and subjected to SDS-PAGE. After transferring and blocking to a polyvinylidene difluoride (PVDF) membrane, the membrane was incubated for 2 h and probed with anti-sirtuin 1 (SIRT1), lipoprotein lipase (LPL), fatty acid synthase (FAS) (Abcam, Cambridge, MA, USA), ATP citrate lyase (ACL) (Millipore, Bedford, MA, USA), phospho-acetyl-CoA carboxylase subtype (p-ACC), and total acetyl-CoA carboxylase subtype (ACC) (Cell Signaling, Danvers, MA, USA) at indicated concentration overnight. After washing and incubation for 2 h with peroxidase-labeled secondary antibody diluted in T-BST, the semaphore was determined by densitometry (Quantity One Analysis software: Bio-Rad).

### 2.6. For Quantitative Reverse Transcription-Polymerase Chain Reaction (qRT-PCR)

qRT-PCR was performed as described previously [21,23]. In brief, five micrograms of the total RNA from adipose tissue were reverse-transcribed with the indicated transcriptase (Invitrogen). PCR was then performed with diluted cDNA, specific primers, and Maxima SYBR Green/Fluorescein qPCR Master Mix (Thermo Scientific, CA, USA). The cycling protocol comprised an initial denaturation step of 10 min at 95 °C with 45 cycles of denaturation for 10 s at 95 °C, followed by annealing for 20 s at 55 °C, and extension for 20 s at 72 °C. LightCycler software was used to detect the threshold cycles (Ct). The relative quantification of gene expression was determined with the comparative Ct method. The averaged Ct was subtracted from the corresponding averaged glyceraldehyde 3-phosphate dehydrogenase (GAPDH) value for each sample. The primers for ACL, acetyl-CoA carboxylase subtype 1 (ACC1), acetyl-CoA carboxylase subtype 2 (ACC2), FAS, LPL, leptin receptors, and GAPDH are listed in Supplementary Table S1.

### 2.7. Statistical Analysis

The effect of the interaction of maternal HF diet and prenatal resveratrol treatment as independent variables was evaluated by Two-way analysis of variance (ANOVA) with Bonferroni correction. The correlation of plasma leptin level and the corresponding body weight of the animal was calculated with Spearman’s rank test. For the variables analysis, outliers which lay 1.5 interquartile ranges (IQRs) above the third quartile or 1.5 IQRs below the first quartile were removed. All statistical tests were performed using SPSS 22.0 for Windows XP (SPSS Inc., Chicago, IL, USA). Values are expressed as mean ± standard error. Statistical significance was defined as *p* < 0.05.

## 3. Results

### 3.1. Maternal Resveratrol Treatment Re-Programmed the Maternal HF Diet Exposure-Induced Visceral Adiposity in the Offspring

**Figure 1:**
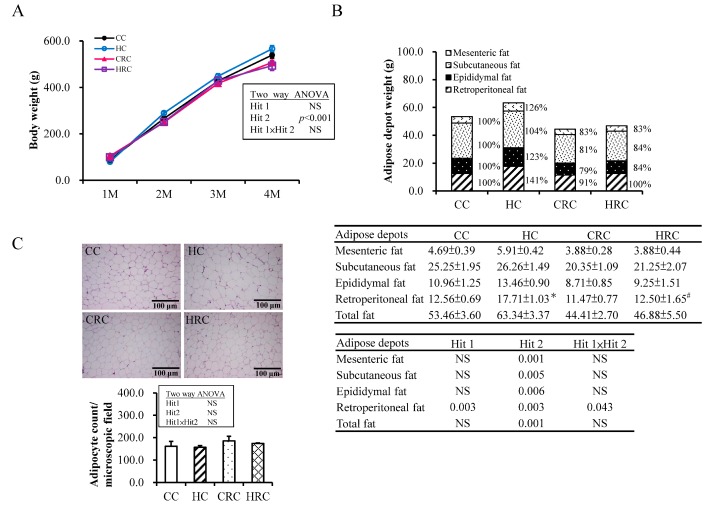
Phenotypes of the offspring groups at four-month-old. (CC: maternal control diet + postnatal control diet; HC: maternal high-fat (HF) diet + postnatal control diet; CRC: maternal control diet + maternal resveratrol treatment + postnatal control diet; HRC: maternal HF diet + maternal resveratrol treatment + postnatal control diet); (**A**) Body weight change with prenatal HF diet exposure or/and resveratrol treatment. The results represent mean ± standard error. Two-way ANOVA analysis result for offspring at four-month-old was shown. (**B**) Weights of adipose depots change with prenatal HF diet exposure or/and resveratrol treatment. The results represent mean ± standard error (* *p* < 0.05 compared to CC group; # *p* < 0.05 compared to HC group). (**C**) Adipocyte sizes change with prenatal HF exposure or/and resveratrol treatment. The bar graph showing the adipocyte counts per microscopic field. (N= CC:14 HC:13 CRC:17 HRC:8).

At first, two-way ANOVA analysis was performed to determine the influences of maternal HF (Hit 1) and maternal resveratrol treatment (Hit 2) on offspring BW and adipose tissues at four-months-old. Figure 1A showed the evolution of BW over time. For BW of offspring at four-months-old, there was a significant main effect of maternal resveratrol therapy on the BW of offspring (F (1, 48): 14.47, *p* < 0.001) without Hit 1/Hit 2 interaction. Maternal resveratrol intervention decreased the BW of adult offspring at four-months-old.

Multiple adipose tissue depots were observed in the offspring. For total adipose tissue, mesenteric, subcutaneous and epidydimal fat depot, only maternal resveratrol treatment had influence, while both prenatal HF diet and maternal resveratrol treatment were important factors contributing toward retroperitoneal adiposity change in the offspring (Figure 1B). Main effects were confirmed for maternal HF diet (F (1, 48) = 9.74, *p* = 0.003) and Hit 2 (F (1, 48) = 10.12, *p* = 0.003). There was a positive interactive effect of H1 and H2 (F (1, 48) = 4.34, *p* = 0.043). The HC group had significantly more retroperitoneal fat tissue than the CC group (17.71 ± 1.03 vs. 12.56 ± 0.69, *p* < 0.001). Maternal resveratrol treatment had significantly decreased the weight of retroperitoneal fat tissue as compared with HC group (HRC vs. HC: 12.50 ± 1.65 vs. 17.71 ± 1.03, *p* = 0.011). H&E staining of offspring adipocytes from retroperitoneal adipose tissue (Figure 1C) revealed a trend showing larger adipocyte in the HC group than in the CC and HRC groups, but without statistical significance. These results suggested that resveratrol intervention during pregnancy showed preventative effects on the visceral adiposity of the offspring with prenatal HF exposure.

### 3.2. Maternal Resveratrol Treatment Reduced the Maternal HF Diet Exposure-Induced Alterations in Liver Enzyme Concentrations

**Figure 2:**
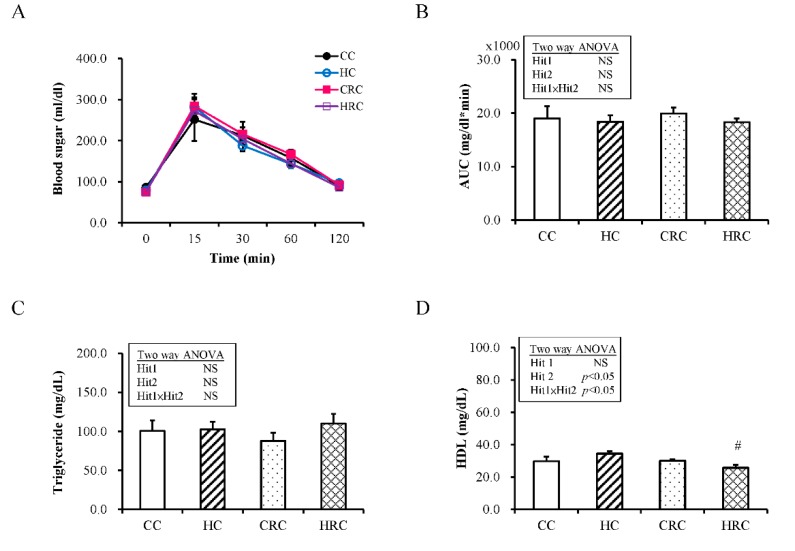
Plasma biochemical analysis of the offspring groups at four-month-old. (**A**) Intraperitoneal glucose tolerance test (IPGTT) (N = CC:6 HC:6 CRC:17 HRC:7); (**B**) Glucose area under the curve (AUC) of IPGTT (N = CC:6 HC:6 CRC:17 HRC:7); (**C**) triglyceride (N = CC:14 HC:13 CRC:17 HRC:8) and (**D**) high-density lipoprotein (HDL) (N = CC:6 HC:6 CRC:6 HRC:6) (CC: maternal control diet + postnatal control diet; HC: maternal high-fat (HF) diet + postnatal control diet; CRC: maternal control diet + maternal resveratrol treatment + postnatal control diet; HRC: maternal HF diet + maternal resveratrol treatment + postnatal control diet) (GPT: glutamic pyruvic transaminase, GOT: glutamic oxaloacetic transaminase, # *p* < 0.05 compared to HC group). All values represent mean ± standard error.

Next, we quantified plasma biochemical assay for the adult offspring. In intraperitoneal glucose tolerance test, there was no effect of maternal HF diet (F (1, 32) = 0.06; *p* = 0.459; Figure 2A and 2B), no effect of maternal resveratrol treatment, and no interaction. The concentration of triglyceride was not affected by Hit 1 (F (1, 48) = 0.95; *p* = 0.334) or Hit 2 (F (1, 48) = 0.04; *p* = 0.834) (Figure 2C). HDL was not affected by Hit 1 alone, (Figure 2D) but the interaction with Hit 2 (F (1, 20) = 5.13, *p* = 0.035) (Hit 1 x Hit 2: F (1, 20) = 5.53, *p* = 0.029) caused a significant decrease of HDL in the HRC group (HC group, 34.50 ± 1.52 mg/dL; HRC group 25.67 ± 1.87 mg/dL, *p* = 0.004). HRC group had lower plasma HDL level than HC group and CRC group had lower plasma HDL level than CC group (Figure 2D). This suggested that maternal resveratrol treatment lead to lower plasma HDL level in offspring.

### 3.3. Maternal Resveratrol Treatment Ameliorated Leptin Dysregulation Induced by Maternal HF Diet Exposure

**Figure 3:**
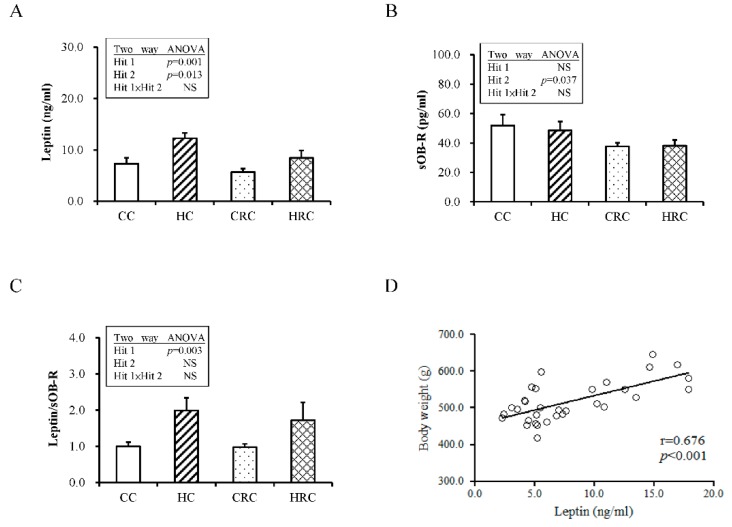
Plasma leptin and soluble leptin receptor (sOB-R) levels with prenatal HF diet exposure or/and prenatal resveratrol treatment (**A**) leptin level (N = CC:12 HC:12 CRC:12 HRC:8) (**B**) sOB-R level (N = CC:12 HC:12 CRC:12 HRC:8) (**C**) leptin/ sOB-R ratio (N = CC:12 HC:12 CRC:12 HRC:8) (**D**) correlation between body weight and plasma leptin level (N = CC:8 HC:8 CRC:8 HRC:8). All values represent mean ± standard error. (CC: maternal control diet + postnatal control diet; HC: maternal high-fat (HF) diet + postnatal control diet; CRC: maternal control diet + maternal resveratrol treatment + postnatal control diet; HRC: maternal HF diet + maternal resveratrol treatment + postnatal control diet).

Leptin, an adipocytokine secreted by adipocytes, has an important regulatory role in obesity and metabolism. Leptin resistance is highly linked to metabolic dysregulation and obesity. We evaluated leptin and soluble leptin receptor (sOB-R) levels in the plasma of the offspring from HF diet-fed dams and the possible de-programming effects induced by maternal resveratrol intervention. First, we measured leptin concentration in the plasma of the offspring. Two-way ANOVA analysis showed a significant main effect for maternal HF diet (Hit 1) (F (1, 40) = 13.06, *p* = 0.001) and maternal resveratrol treatment (Hit 2) (F (1, 40) = 6.69, *p* = 0.013), but without Hit 1/ Hit 2 interaction (Figure 3A). Plasma leptin level was higher in the HC group than the CC group. With resveratrol treatment, the plasma leptin level of the offspring in the HRC group significantly decreased. Thereafter, we determined the concentration of plasma sOB-R and found only the effect of maternal resveratrol treatment was significant (F (1, 40) = 4.68; * *p* = 0.037; Figure 3B), but no effect of maternal HF diet (F (1, 40) = 0.072; *p* = 0.790) or interaction of the effects were observed. Total leptin to sOB-R concentration ratio acts as a biomarker of leptin resistance [24,25,26], therefore total leptin/sOB-R ratio was determined. Two-way ANOVA analysis showed a significant main effect for maternal HF diet (F (1, 40) = 9.76, *p* = 0.003) rather than for maternal resveratrol therapy and there was no Hit 1/Hit 2 interaction (Figure 3C). This ratio was higher in the offspring that received prenatal HF diet than the offspring from dams that received prenatal control diet. Correlation between body weight and circulating leptin is presented in Figure 3D. It seems that plasma leptin levels reflect the amount of adipose tissue.

Moreover, whether leptin receptor levels in the adipose tissue of offspring is altered by prenatal HF intake and maternal resveratrol treatment was then determined. The expression levels of mRNA of short form of leptin receptor (OBRa) and long form of leptin receptor (OBRb) in retroperitoneal adipose depot were tested using qPCR. Two-way ANOVA analysis showed a significant main effect of maternal resveratrol treatment for OBRa mRNA expression (F (1, 40) = 20.34, *p* < 0.001) without Hit 1 and Hit 2 interaction (Supplementary Figure S2A). Gene expression of OBRb was not significantly affected by Hit 1 alone, (Supplementary Figure S2A, but the interaction of maternal resveratrol (F (1, 40) = 11.31, *p* = 0.002) caused a significant drop of OBRb gene expression in the HRC group (Supplementary Figure S2B), while there was no significant difference for the gene expression of leptin in retroperitoneal depot among the four groups (Supplementary Figure S2C).

### 3.4. Maternal Resveratrol Treatment Reduced Maternal HF Diet Exposure-Induced Adiposity in the Offspring via a SIRT1-Independent Mechanism

**Figure 4:**
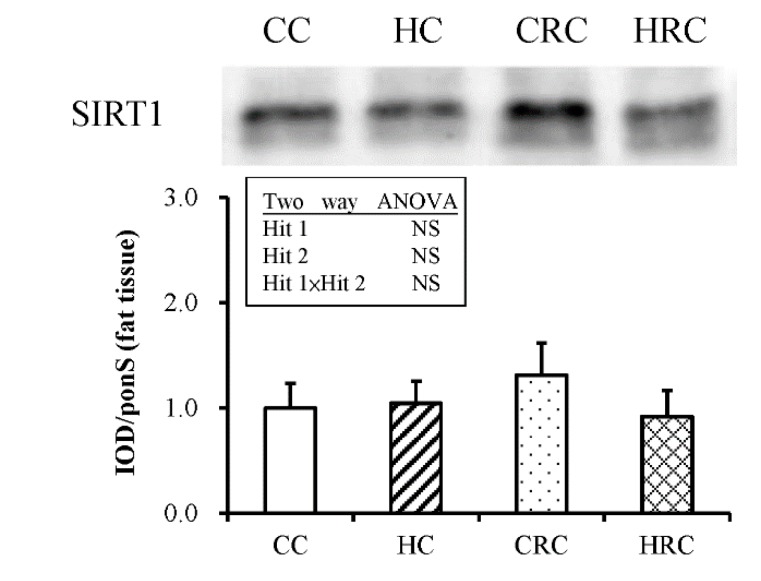
Sirtuin-1 (SIRT1) abundance in retroperitoneal adipose tissue of offspring with prenatal HF diet exposure or/and prenatal resveratrol therapy. All values represent mean ± standard error. (CC: maternal control diet + postnatal control diet; HC: maternal high-fat (HF) diet + postnatal control diet; CRC: maternal control diet + maternal resveratrol treatment + postnatal control diet; HRC: maternal HF diet + maternal resveratrol treatment + postnatal control diet). (N = CC:10 HC:10 CRC:10 HRC:10).

SIRT1 is an essential metabolic and regulatory transcription factor. Resveratrol is an activator of SIRT1. Previously, we found that resveratrol administration to the offspring could improve fatty acid metabolism and reduce obesity by activating SIRT1 from retroperitoneal fat tissues [19]. In this study, we wanted to examine whether prenatal administration of resveratrol could activate SIRT1 in the offspring. We analyzed the abundance of SIRT1 from the retroperitoneal fat tissue of offspring by western blotting and found that there was again no effect of Hit 1 (F (1, 36) = 0.47; *p* = 0.498), no effect of B (F (1, 36) = 0.132; *p* = 0.718), and no interaction of the effects (Figure 4). Therefore, maternal resveratrol administration could be considered to improve adiposity and leptin dysregulation in offspring by a mechanism that was independent of SIRT1.

### 3.5. Administration of Resveratrol during Pregnancy Altered the Expression of Genes Crucial for Fatty Acid Synthesis in the Offspring

Adipocytes are special cells that deposit superfluous energy as triglycerides (TG) in lipid droplets, a process known as lipogenesis. When energy is needed, the stored TG is hydrolyzed by activating the pathway involved in lipolysis. Both lipolysis and lipogenesis are highly complex cellular processes that are under tight regulation. We showed that administration of resveratrol to pregnant dams can improve visceral adiposity in offspring. We studied the genetic expression of several important enzymes for fatty acid metabolism in the retroperitoneal fat tissue of offspring by qRT-PCR and found that prenatal HF diet exposure or prenatal resveratrol therapy had no influence on the expression of genes that produce lipoprotein lipase (LPL) in the retroperitoneal fat tissue (Supplementary Figure S3).

**Figure 5:**
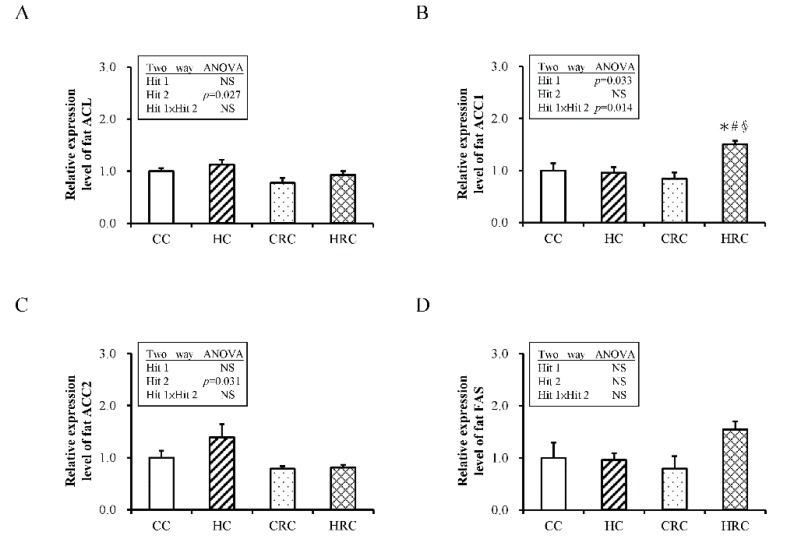
The expression of genes in retroperitoneal adipose tissue of the offspring at four-month-old with prenatal HF diet exposure or/and prenatal resveratrol treatment. (**A**) ATP citrate lyase (ACL), (**B**) Acetyl-CoA carboxylase 1 (ACC1), (**C**) Acetyl-CoA carboxylase 2 (ACC2), and (**D**) Fatty acid synthase (FAS). All values represent mean ± standard error. (CC: maternal control diet + postnatal control diet; HC: maternal high-fat (HF) diet + postnatal control diet; CRC: maternal control diet + maternal resveratrol treatment + postnatal control diet; HRC: maternal HF diet + maternal resveratrol treatment + postnatal control diet). (* *p* < 0.05 compared to CC group; # *p* < 0.05 compared to HC group; & *p* < 0.05 compared to CRC group). (N = CC:12 HC:12 CRC:12 HRC:8).

**Figure 6:**
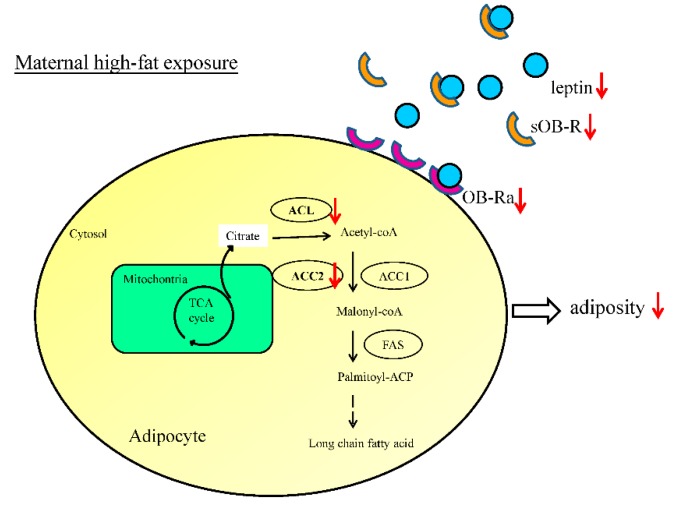
A schematic illustration showing how maternal resveratrol alters the levels of enzymes required for fatty acid synthesis.

For gene expressions of lipogenesis related enzymes, there was a marked effect of maternal resveratrol administration on the expression of ACL mRNA (F (1, 40) = 5.31; *p* = 0.027), but no influence of maternal HF diet was present (F (1, 40) = 2.38; *p* = 0.131), and there was no interaction (F (1, 40) = 0.10; *p* = 0.750). Expression of ACC1 mRNA was not significantly influenced by maternal resveratrol treatment alone, but the interaction of maternal HF diet (F (1, 40) = 4.89, *p* = 0.033) caused a marked increase of ACC1 mRNA in HRC group. Only the effect of maternal resveratrol therapy was significant (F (1, 40) = 5.01; *p* < 0.031; Figure 5C), but no effect of Hit 1 (F (1, 40) = 1.05; *p* = 0.311) or interaction of the effects were observed. There was no effect of Hit 1 on FAS mRNA expression (F (1, 40) = 2.42; *p* = 0.128; Figure 5D), no effect of B (F (1, 40) = 0.368; *p* = 0.548), and no interaction of these effects. In summary, treatment with resveratrol during pregnancy and lactation period reduced the gene expression of ACL and ACC2 in the offspring (Figure 5 and Figure 6). Western blot also demonstrated a trend for higher FAS abundance in HRC group than CC group.

## 4. Discussion

In this study, we found that maternal resveratrol treatment can reduce maternal HF diet/obesity-induced retroperitoneal adiposity plus leptin dysregulation in the offspring. Maternal resveratrol treatment during pregnancy and lactation could reduce the gene expression of *ACL* and *ACC2* of retroperitoneal depot in offspring. Prenatal resveratrol therapy has a considerably positive influence on metabolic mechanisms of visceral adipose tissue in the rat offspring.

In contrast to the short-term (postnatal 21 days old) effects published by Ros P. et al. [27], our study illustrated a long-term (4-month-old) effect of prenatal resveratrol. For the short-term effects of maternal resveratrol treatment, Ros P. et al., showed maternal resveratrol intake during pregnancy and lactation decreased maternal HF induced BW and visceral adiposity of offspring. Maternal resveratrol intake increased blood glucose of offspring. While maternal resveratrol treatment did not change the elevated blood leptin, leptin mRNA and LPL mRNA expressions of visceral adipose tissue of offspring induced by maternal HF. For long-term effects of maternal resveratrol treatment, our study showed that prenatal resveratrol intake also can decrease maternal HF induced BW and visceral adiposity of offspring. Maternal resveratrol treatment also did not change the leptin mRNA and LPL mRNA expressions of visceral adipose tissue of offspring that was induced by maternal HF. In contrast to short-term effect, maternal resveratrol did not induce the elevation of blood sugar at 4-month-old. Although maternal resveratrol therapy had no effect on the plasma leptin level at 21-day-old [18], we found maternal resveratrol therapy decreased the high plasma leptin level in offspring induced by maternal HF at 4-month-old. However, different diet and animals were used by Ros P. et al., and further studies are needed to make a conclusion. Regarding the inconsistency between plasma leptin level and leptin mRNA expression of retroperitoneal depot, test for leptin mRNA expression in other fat deposits may explain this phenomenon.

In this study, only male animals were used because obesity, hypertension, and related metabolic disorders occur at a higher rate than females [27,28]. Since some effects of developmental programming on long-term offspring health are gender-specific, further studies to investigate the gender difference of prenatal insults in the future are needed. Besides, since retroperitoneal fat has been shown to be related to more metabolic profiles than other fat depots [29], retroperitoneal fat depot was selected for this study. It is interesting that prenatal resveratrol therapy reduced plasma HDL level of offspring. Further study to determine LDL level and the HDL/LDL ratio may provide more information.

We found that maternal HF diet and obesity lead to leptinemia but does not influence plasma sOB-R levels in the offspring. Maternal resveratrol treatment was able to reduce plasma sOB-R and leptin levels. Leptin, secreted by the adipose tissue, regulates energy balance via relevant receptors in the hypothalamus, and suppresses appetite that eventually diminishes fat storage [30]. Although the hypothalamus–adipose tissue axis majorly regulates energy homeostasis through leptin, other peripheral organs are also known to be involved [31]. For example, leptin can modulate lipogenesis in the adipose tissue by regulating the levels of lipogenic enzymes [32]. Although circulatory blood levels of leptin reduce the desire for food consumption, obese individuals generally display leptin resistance with a higher circulatory leptin level but impaired receptor sensitivity compared with optimal weight individuals [33]. In addition to central leptin resistance that develops in the nervous system, peripheral leptin resistance may also develop in muscles, liver, and adipose tissue [34]. In human and animal studies, subjects with obesity showed a lower leptin receptor mRNA level in adipose depots than lean subjects [35].

Leptin in circulation exists as a free form and a bound form (bound to soluble receptor in plasma) [36]. sOB-R is the extracellular cleaved part of the membrane leptin receptor, and it can modulate the bioavailability of circulatory leptin [24,37]. sOB-R level is regulated by plasma leptin concentration, lipotoxicity, and endoplasmic reticulum stress; a disturbance in its level causes several metabolic conditions and neoplasms [37,38,39,40,41,42,43]. The ratio of total leptin and sOB-R concentrations is a biomarker of leptin resistance [24,25,26]. It is associated with metabolic disorders and is recognized as a predictor of early type II diabetes [39]. Adipose mass loss in obese adults tends to balance the ratio of leptin to sOB-R, suggesting amelioration of leptin resistance following weight loss [44]. In our previous study, we found that resveratrol therapy can decrease the plasma leptin levels and increase leptin receptor expression in retroperitoneal adipose tissue of animals with prenatal and postnatal HF diet exposure [17]. In this study, we found that resveratrol treatment in dams with HF diet/obesity can improve leptin dysregulation of the offspring by decreasing leptin/sOB-R ratio. This de-programming effect of prenatal resveratrol treatment upon maternal HF diet exposure is mainly because of a decrease in the plasma leptin level in the offspring.

Through lipogenesis and lipolysis, the adipose tissue stores triglycerides and releases fatty acids establishing homeostasis. Lipogenic enzymes of the adipose tissue are key targets of leptin [45]. ACC1, ACC2, ACL, FAS, and LPL are all crucial enzymes for lipid metabolism. To study their relevance in programming and de-programming of adiposity by maternal HF diet and maternal resveratrol therapy, respectively, the gene expression of these enzymes was investigated. We found that maternal resveratrol treatment during pregnancy and lactation can modulate the expression of enzymes related to fatty acid synthesis in the offspring, i.e., decrease the expression of *ACL* and *ACC2*. In mammalian cells, fatty acids are formed from carbohydrates in the liver and adipose tissue. Glucose is converted into pyruvate, which is further converted to citrate in the mitochondria. Citrate is transported into the cytosol and cleaved into acetyl-CoA and oxaloacetate by ACL. Acetyl-CoA is then carboxylated into malonyl-CoA by ACC. Then, FAS condensates malonyl-CoA and acetyl-CoA into the long-chain fatty acid, saturated palmitate [46]. ACL is a master enzyme involved in de novo fatty acid synthesis that can also be activated by insulin [47]. There are two isoforms of ACC, ACC1 and ACC2. Both these are encoded by different genes and have distinct cellular localization: ACC1 is located in the cytosol, and ACC2 in the mitochondrial membrane [48]. ACC1 is highly expressed in the liver and adipose tissue and its function is influenced by diet [48,49]. For example, a carbohydrate-rich, low-fat diet can stimulate the expression and activities of ACC1. ACC2 is the major form of carboxylase in the skeletal muscle and heart. It regulates mitochondrial fatty acid oxidation by its product malonyl-CoA, via the inhibition of carnitine palmitoyl transferase 1 [50]. The contrasting gene expression pattern of *ACC1* and *ACC2* has also been reported by others. Kreuz et al. showed that in Zucker diabetic rats, the expression of ACC2 is significantly increased and ACC1 is reduced in oxidative tissues, compared with Zucker lean control rats [50]. Therefore, the effects of prenatal resveratrol on fatty acid synthesis-related enzymes may support its re-programming mechanism against maternal HF diet/obesity.

We previously found that resveratrol treatment in the offspring can improve the maternal and postnatal high fat diet-induced decreased expression of SIRT1 in the retroperitoneal adipose tissue [17]. However, we found that prenatal resveratrol treatment in pregnant dams on HF diet does not activate SIRT1 expression in the retroperitoneal adipose depot of the offspring. This may be because prenatal HF diet, unlike prenatal HF diet or maternal obesity combined with postnatal HF diet, may not notably decrease SIRT1 expression in the offspring. Prenatal resveratrol intervention does not over-activate SIRT1 under normal circumstances. Thus, the re-programming effects of prenatal resveratrol treatment on maternal HF diet exposure may be independent of SIRT1. SIRT-1 independent manner evoked by resveratrol has also been reported [51,52,53]. In addition to SIRT1, resveratrol may act through other mechanisms to ameliorate the symptoms of metabolic syndrome and related disorders, such as AMP-activated protein kinase (AMPK), Nuclear factor (erythroid-derived 2)-like 2 (Nrf2), Renin-Angiotensin System, and the nuclear factor kappa-B cells (NF-κB) family of proteins [15]. The precise mechanism for prenatal resveratrol treatment to achieve its re-programming effect for maternal HF diet exposure needs further investigation.

## 5. Conclusions

Leptin dysregulation occurs in the offspring with maternal HF diet and obesity. Exposure to prenatal HF diet alters lipogenesis, which may further promote adiposity in the offspring. Our results suggest that maternal resveratrol administration has beneficial effects on adiposity and leptin dysregulation in the male rat offspring affected by prenatal HF diet exposure and maternal obesity. Maternal resveratrol treatment shows promising potential in reducing the negative effects of maternal HF diet related obesity on the offspring.

## Supplementary Materials

The following are available online at https://www.mdpi.com/1660-4601/17/8/2780/s1, Figure S1. The body weight changes of dam on regular diet and high-fat (HF) diet. (N = 10 for control diet and N = 10 for HF diet), Figure S2. The expression of genes in retroperitoneal adipose tissue of the offspring with prenatal HF diet exposure or/and prenatal resveratrol treatment. (A) short-form of leptin receptor (OBRa), (B) long-form of leptin receptor (OBRb), (C) leptin. All values represent mean ± standard error. (CC: maternal control diet + postnatal control diet; HC: maternal high-fat (HF) diet + postnatal control diet; CRC: maternal control diet + maternal resveratrol treatment + postnatal control diet; HRC: maternal HF diet + maternal resveratrol treatment + postnatal control diet). (* *p* < 0.05 compared to CC group; # *p* < 0.05 compared to HC group; & *p* < 0.05 compared to CRC group, mean ± standard error).(N = CC:12 HC:12 CRC:12 HRC:8), Figure S3. The expression of lipoprotein lipase (LPL) in retroperitoneal adipose tissue of the offspring with prenatal HF diet exposure or/and prenatal resveratrol treatment., Figure S4. The abundance of proteins in retroperitoneal adipose tissue of the offspring with prenatal HF diet exposure or/and prenatal resveratrol treatment. (A) ATP citrate lyase (ACL) (N = CC:8 HC:8 CRC:8 HRC:8), (B) Phospho- acetyl-CoA carboxylase (p-ACC)/ Acetyl-CoA carboxylase (ACC) (N = CC:8 HC:8 CRC:8 HRC:8), (C) Fatty acid synthase (FAS) (N = CC:10 HC:10 CRC:10 HRC:10), and (D) Lipoprotein lipase (LPL). All values represent mean ± standard error. (N = CC:5 HC:5 CRC:5 HRC:5) (CC: maternal control diet + postnatal control diet; HC: maternal high-fat (HF) diet + postnatal control diet; CRC: maternal control diet + maternal resveratrol treatment + postnatal control diet; HRC: maternal HF diet + maternal resveratrol treatment + postnatal control diet). (* *p* < 0.05)., Table S1: Primer sequence.
