# Supplementary material for: Maternal Resveratrol Treatment Re-Programs and Maternal High-Fat Diet-Induced Retroperitoneal Adiposity in Male Offspring
Source: Int J Environ Res Public Health. 2020 Apr 17;17(8):2780. doi: 10.3390/ijerph17082780 (PMC7215689; doi:10.3390/ijerph17082780)
Supplement: Supplementary file 1 [file ijerph-17-02780-s001.zip › ijerph-761894 supplementary.docx]

**Supplementary Material**

Article

Maternal Resveratrol Treatment Re-Programs Maternal High-fat diet-induced Retroperitoneal Adiposity in Male Offspring

Ti-An Tsai ^1^†, Chang-Ku Tsai ^1^†, Li-Tung Huang ^1^, Jiunn-Ming Sheen ^1^, Mao-Meng Tiao ^1^, You-Lin Tain ^1^, Chih-Cheng Chen ^1^, I-Chun Lin ^1^, Yun-Ju Lai ^2^, Ching-Chou Tsai ^2^,
Yu-Ju Lin ^2^ and Hong-Ren Yu ^1,^*

^1^ Department of Pediatrics, Chang Gung Memorial Hospital-Kaohsiung Medical Center; Graduate Institute of Clinical Medical Science, Chang Gung University College of Medicine, Kaohsiung, Taiwan; tiantsai@cgmh.org.tw (T.-A.T.); wind518@cgmh.org.tw (C.-K.T.); litung.huang@gmail.com (L.-T.H.); ray.sheen@gmail.com (J.-M.S.); pc006581@yahoo.com.tw (M.-M.T.); tainyl@hotmail.com (Y.-L.T.); charllysc@adm.cgmh.org.tw (C.-C.C.)

^2^ Department of Obstetrics and Gynecology, Chang Gung Memorial Hospital-Kaohsiung Medical Center; uc22@adm.cgmh.org.tw (I.-C.L.); lusionbear@hotmail.com (Y.-J.L.); nickcctsai@yahoo.com.tw (C.-C.T.); lyu015@cgmh.org.tw (Y.-J.L.)

***** Correspondence: yuu2004taiwan@yahoo.com.tw; Tel.: +886-7-731-7123 ext. 8713;
Fax.: +886-7-733-8009

†Equal contribution

**Table S1.** Primer sequence.

| Rat ACL | Forward | 5'-ACCCAGAGGAAGCCTACATTGC-3' |
| --- | --- | --- |
|  | Reverse | 5'-TTCGCCAGTTCGTTGACACC-3' |
| Rat ACC1 | Forward | 5'-TGAGGAGGACCGCATTTATC-3' |
|  | Reverse | 5'-GCATGGAATGGCAGTAAGGT-3' |
| Rat ACC2 | Forward | 5'-CGCTGCGGTCAAGTGT-3' |
|  | Reverse | 5'-CGTTGGCGTAGTTGTTATT-3' |
| Rat FAS | Forward | 5'-AGATCCTGGAACGTGAACATGA-3' |
|  | Reverse | 5'-GCCGTACTTCACGAATGGGT-3' |
| Rat OBRa | Forward | 5'-ACACTGTTAATTTCACACCAGAG-3' |
|  | Reverse | 5'-AGTCATTCAAACCATAGTTTAGG-3' |
| Rat OBRb | Forward | 5'-TCTTCTGGAGCCTGAACCCATTTC-3' |
|  | Reverse | 5'-TTCTCACCAGAGGTCCCTAAACT-3' |
| Rat LPL | Forward | 5'-GTACAGTCTTGGAGCCCATGC-3' |
|  | Reverse | 5'-GCCAGTAATTCTATTGACCTTCTTGTT-3' |
| Rat GAPDH | Forward | 5'-TCTTGTGCAGTGCCAGCCTC-3' |
|  | Reverse | 5'-GTCACAAGAGAAGGCAGCCCTGG-3' |

ACC1 (Acetyl-CoA carboxylase 1)

ACC2 (Acetyl-CoA carboxylase 2)

ACL (ATP citrate lyase)

FAS (Fatty acid synthase)

LPL (Lipoprotein lipase)

OBRa (Short form of leptin receptor)

OBRb (Long form of leptin receptor)

SIRT-1 (NAD-dependent deacetylase sirtuin-1)

GAPDH (Glyceraldehyde 3-phosphate dehydrogenase)
