# Supplementary figures and images for: Maternal Resveratrol Treatment Re-Programs and Maternal High-Fat Diet-Induced Retroperitoneal Adiposity in Male Offspring
Source: Int J Environ Res Public Health. 2020 Apr 17;17(8):2780. doi: 10.3390/ijerph17082780 (PMC7215689; doi:10.3390/ijerph17082780)

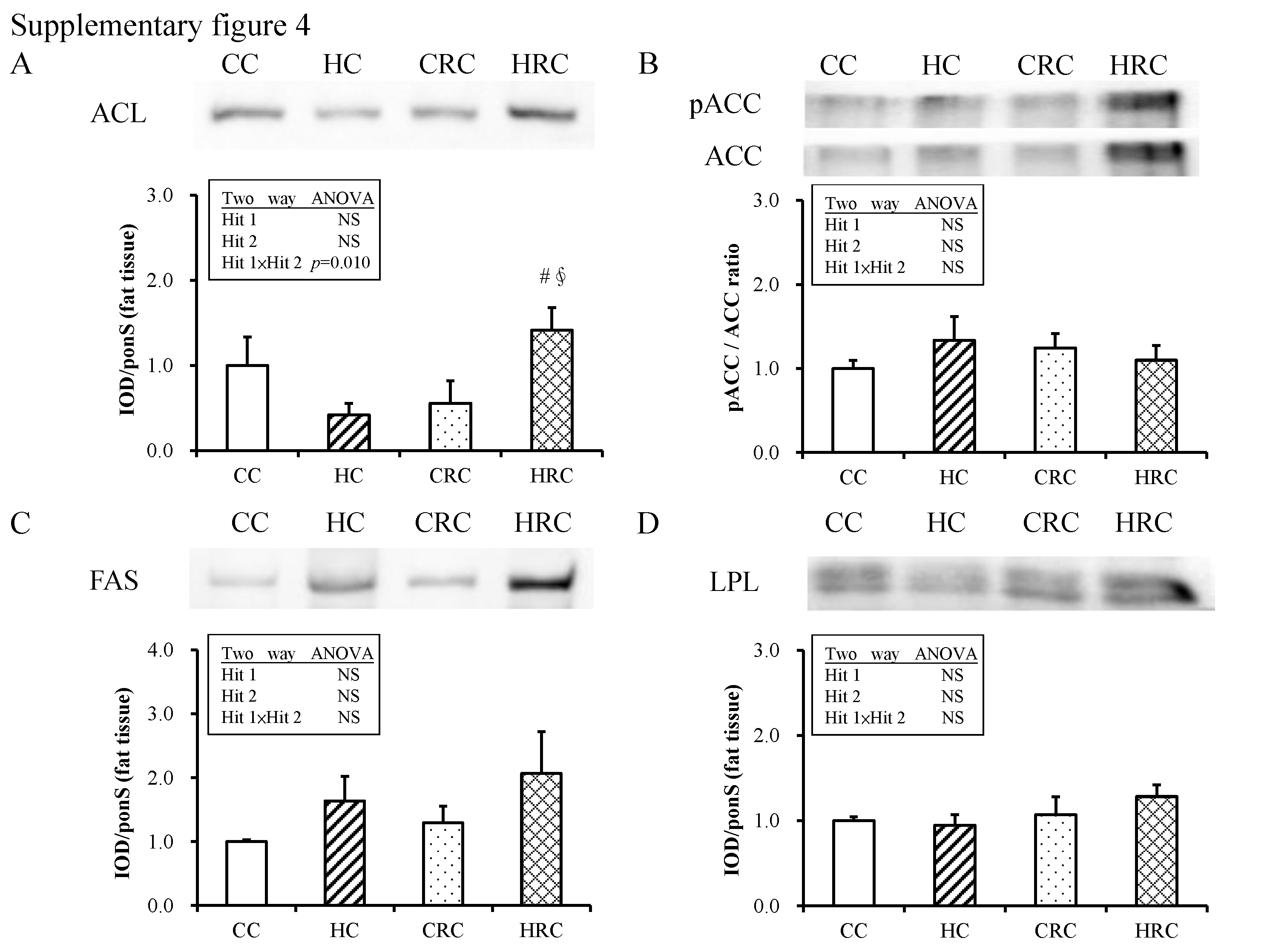

Supplement: Supplementary file 1 [file ijerph-17-02780-s001.zip › Sup Figure 4.tiff]

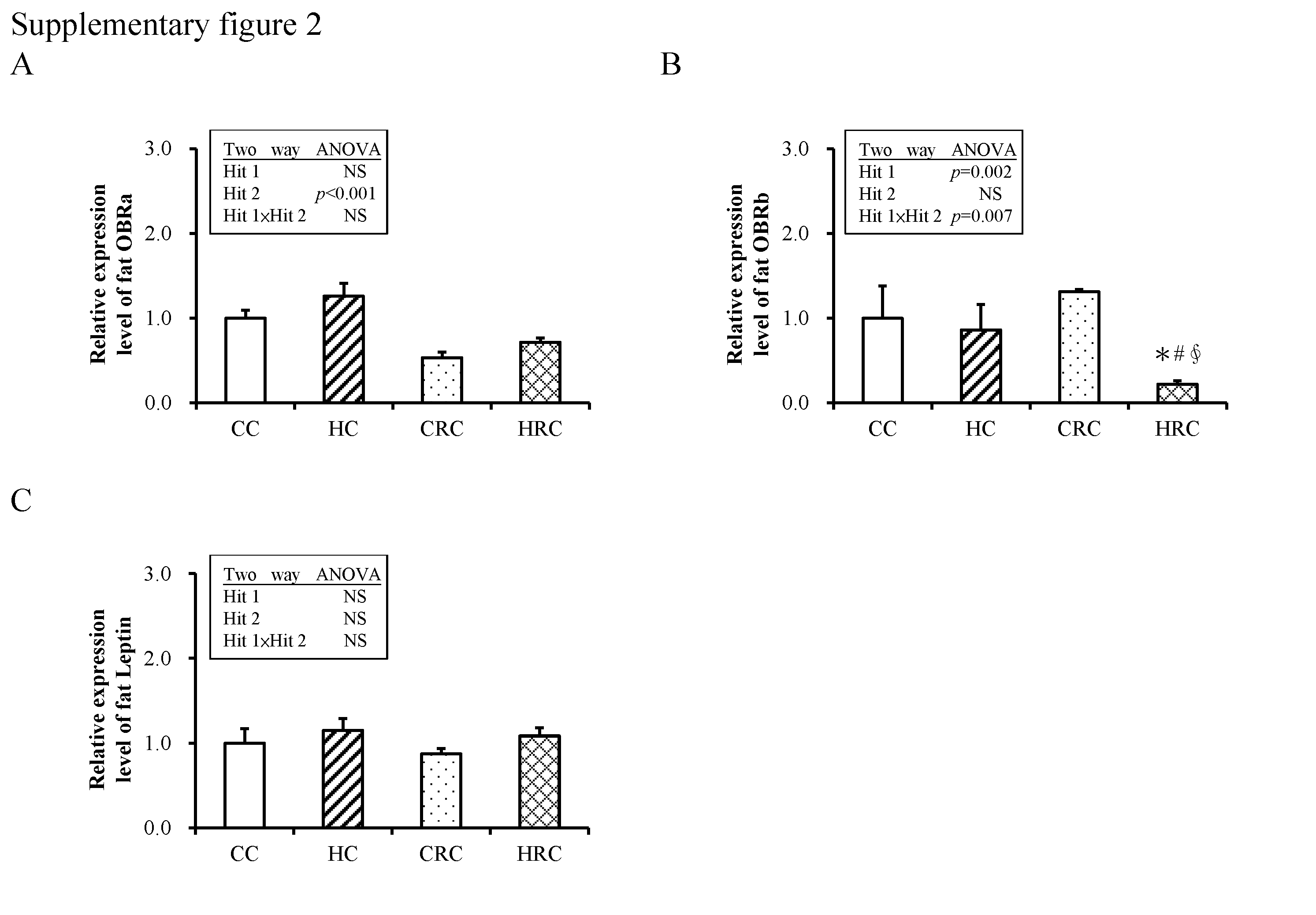

Supplement: Supplementary file 1 [file ijerph-17-02780-s001.zip › Sup Figure 2.tiff]

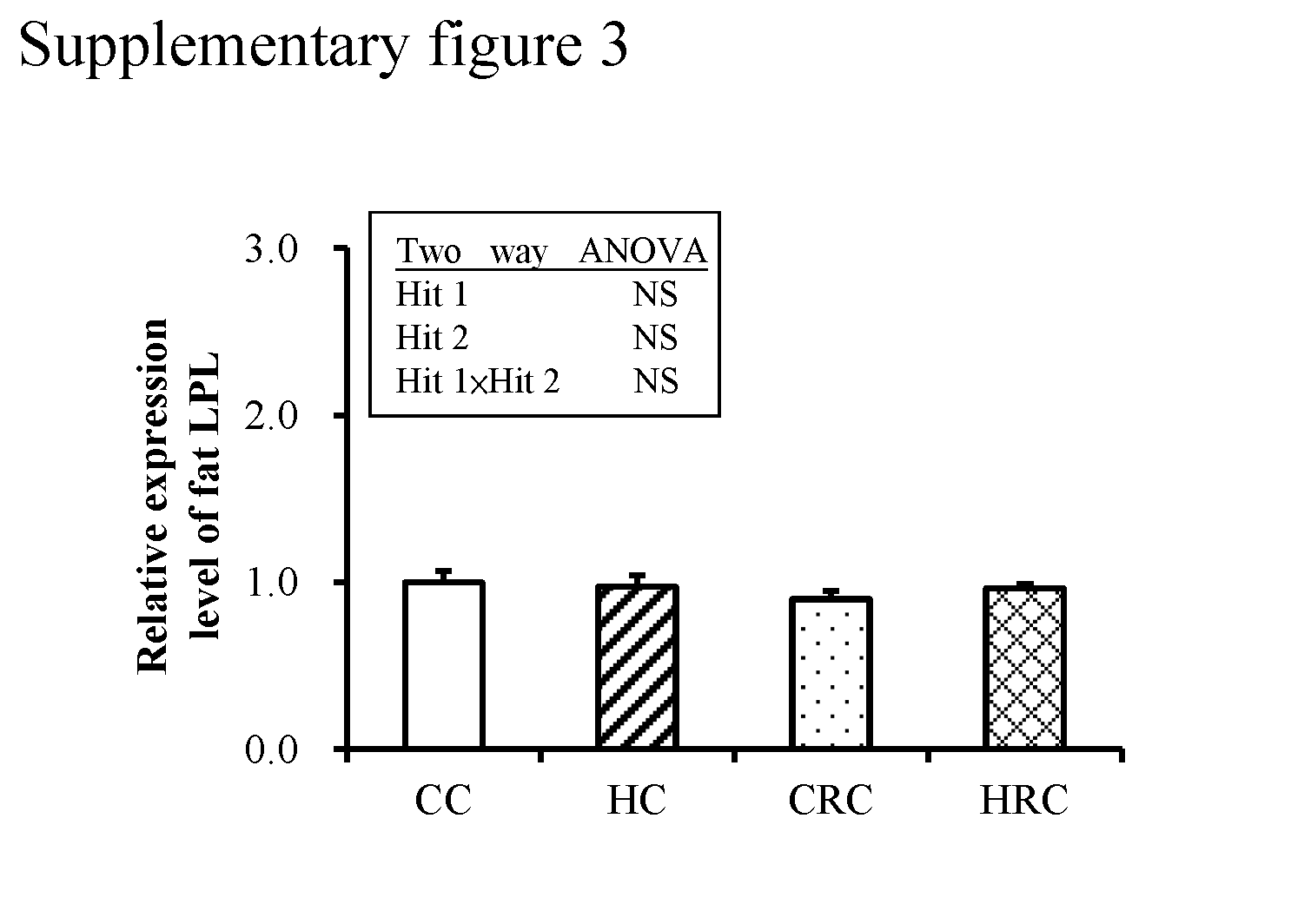

Supplement: Supplementary file 1 [file ijerph-17-02780-s001.zip › Sup Figure 3.tiff]
