# Supplementary material for: Maternal Resveratrol Treatment Re-Programs and Maternal High-Fat Diet-Induced Retroperitoneal Adiposity in Male Offspring
Source: Int J Environ Res Public Health. 2020 Apr 17;17(8):2780. doi: 10.3390/ijerph17082780 (PMC7215689; doi:10.3390/ijerph17082780)
Supplement: Supplementary file 1 [file ijerph-17-02780-s001.zip › Supplementary table 1.doc]

Supplementary Table 1.

Primer sequence

| Rat ACL | Forward | 5'-ACCCAGAGGAAGCCTACATTGC-3' |
| --- | --- | --- |
|  | Reverse | 5'-TTCGCCAGTTCGTTGACACC-3' |
| Rat ACC1 | Forward | 5'-TGAGGAGGACCGCATTTATC-3' |
|  | Reverse | 5'-GCATGGAATGGCAGTAAGGT-3' |
| Rat ACC2 | Forward | 5'-CGCTGCGGTCAAGTGT-3' |
|  | Reverse | 5'-CGTTGGCGTAGTTGTTATT-3' |
| Rat FAS | Forward | 5'-AGATCCTGGAACGTGAACATGA-3' |
|  | Reverse | 5'-GCCGTACTTCACGAATGGGT-3' |
| Rat OBRa | Forward | 5'-ACACTGTTAATTTCACACCAGAG-3' |
|  | Reverse | 5'-AGTCATTCAAACCATAGTTTAGG-3' |
| Rat OBRb | Forward | 5'-TCTTCTGGAGCCTGAACCCATTTC-3' |
|  | Reverse | 5'-TTCTCACCAGAGGTCCCTAAACT-3' |
| Rat LPL | Forward | 5'-GTACAGTCTTGGAGCCCATGC-3' |
|  | Reverse | 5'-GCCAGTAATTCTATTGACCTTCTTGTT-3' |
| Rat GAPDH | Forward | 5'-TCTTGTGCAGTGCCAGCCTC-3' |
|  | Reverse | 5'-GTCACAAGAGAAGGCAGCCCTGG-3' |

ACC1 (Acetyl-CoA carboxylase 1)

ACC2 (Acetyl-CoA carboxylase 2)

ACL (ATP citrate lyase)

FAS (Fatty acid synthase)

LPL (Lipoprotein lipase)

OBRa (Short form of leptin receptor)

OBRb (Long form of leptin receptor)

SIRT-1 (NAD-dependent deacetylase sirtuin-1)

GAPDH (Glyceraldehyde 3-phosphate dehydrogenase)
